# Extracellular Vesicle-Derived miR-105-5p Promotes Malignant Phenotypes of Esophageal Squamous Cell Carcinoma by Targeting SPARCL1 *via* FAK/AKT Signaling Pathway

**DOI:** 10.3389/fgene.2022.819699

**Published:** 2022-03-03

**Authors:** Binjun He, Kang Zhang, Xiaoliang Han, Chao Su, Jiaming Zhao, Guoxia Wang, Guzong Wang, Liuya Zhang, Wenbin Hu

**Affiliations:** ^1^ Department of Thoracic Surgery, Shaoxing People’s Hospital (Zhejiang University School of Medicine), Shaoxing, China; ^2^ Department of Thoracic Surgery, Affiliated Hospital of Shaoxing University /Shaoxing Municipal Hospital, Shaoxing, China

**Keywords:** EVs, miR-105-5p, SPARCL1, ESCC, FAK/AKT, proliferation, migration, invasion

## Abstract

**Objective:** Esophageal squamous cell carcinoma (ESCC) presents high morbidity and mortality. It was demonstrated that blood-derived vesicles can facilitate ESCC development and transmit regulating signals. However, the molecular mechanism of vesicle miRNA secreted by tumor cells affecting ESCC progression has not been explored.

**Methods:** The mRNA-related signaling pathways and differentially expressed genes were screened out in TCGA dataset. The levels of miRNA-105-5p and SPARCL1 were determined by qRT-PCR. Protein level determination was processed using Western blot. The interaction between the two genes was verified with the dual-luciferase method. A transmission electron microscope was utilized to further identify extracellular vesicles (EVs), and co-culture assay was performed to validate the intake of EVs. *In vitro* experiments were conducted to evaluate cell function changes in ESCC. A mice tumor formation experiment was carried out to observe tumor growth *in vivo*.

**Results:** MiRNA-105-5p expression was increased in ESCC, while SPARCL1 was less expressed. MiRNA-105-5p facilitated cell behaviors in ESCC through targeting SPARCL1 and regulating the focal adhesion kinase (FAK)/Akt signaling pathway. Blood-derived external vesicles containing miRNA-105-5p and EVs could be internalized by ESCC cells. Then, miRNA-105-5p could be transferred to ESCC cells to foster tumorigenesis as well as cell behaviors.

**Conclusion:** EV-carried miRNA-105-5p entered ESCC cells and promoted tumor-relevant functions by mediating SPARCL1 and the FAK/Akt signaling pathway, which indicated that the treatment of ESCC *via* serum EVs might be a novel therapy and that miRNA-105-5p can be a molecular target for ESCC therapy.

## Introduction

Esophageal carcinoma (EC) ranks sixth in mortality (544,000 deaths) and seventh in incidence (604,000 new cases) in 2020 (Sung et al., 2021), which means that EC is a serious threat to human health. Esophageal squamous cell carcinoma (ESCC) makes up the majority of EC (Lin et al., 2013). ESCC is formed by the abnormal proliferation of esophagus squamous epithelium, which is a kind of cancer with unobvious early clinical features. Besides, most ESCC patients are diagnosed at advanced stages accompanied by distant metastasis, and their 5-year survival rates are lower than 20% after receiving conventional therapies (Siegel et al., 20182018; Sawada et al., 2019). Accordingly, finding effective therapeutic approaches for ESCC patients is of substantial importance to the enhancement of advanced ESCC patients’ survival.

With deeper investigations of miRNAs in recent years, it has been confirmed that miRNAs can mediate the growth and metastasis of multiple cancers (Xu et al., 2018; Sheng et al., 2019; Wu et al., 2021). For instance, miRNA-10b-3p is capable of fostering ESCC growth and metastasis (Priniski et al., 2019). MiRNA-133b can inhibit ESCC cell processes (Zeng et al., 2019). MiRNA-105-5p is a member of miRNAs, yet there have been relatively few studies about its role in cancers. Currently, it has only been reported that miRNA-105-5p modulates PES1 in liver cancer stem cells to facilitate cell growth (Wei et al., 2019). Nonetheless, there has been no research on the relationship between miRNA-105-5p and ESCC. To this end, the present study aimed to gain deeper insight into the regulatory mechanism.

Extracellular vesicles (EVs) are extracellular membrane particles’ component with diameters of 40–1,000 nm, whose outer layer is a bilayer lipid membrane while the inside encapsulates different proportions of DNA, RNA, and protein components (Jin et al., 2021). Numerous reports indicate that EVs are usually existing in body fluids, including cerebrospinal fluid, blood, urine, saliva, pleural effusion, and ascites (Caivano et al., 2015). EVs have many biological functions, such as intercellular information exchange, protecting and repairing damaged cells and tissue, participating in immune response, and promoting angiogenesis (Abels and Breakefield, 2016; Xu et al., 2016). Recently, research has suggested that serum EVs can transfer miRNAs to tumor cells to regulate tumorigenesis. For example, exosomal miRNA-660-5p is sent to foster the metastasis of non-small cell lung cancer (NSCLC) cells (Qi et al., 2019). MiRNA-21 carried on Evs can be transferred to ESCC cells to induce ESCC cell proliferation (Tanaka et al., 2013). Nevertheless, whether miRNA-105-5p in Evs could modulate ESCC remains unknown.

To conclude, we confirmed that serum EV-derived miRNA-105-5p could be transferred to ESCC cells to foster the progression of ESCC cells by regulating the focal adhesion kinase (FAK)/Akt signaling pathway, indicating that miRNA-105-5p might be a novel signal facilitating ESCC development.

## Materials and Methods

### Bioinformatics Methods

ESCC-related miRNA chip data GSE55856 (normal: *n* = 108, tumor: *n* = 108) were accessed from the GEO database, and differential analysis was performed to screen differentially expressed miRNAs (DEmiRNAs) using the “limma” package (|logFC|>2, *p*adj < 0.01). Meanwhile, the ESCC-related miRNA dataset (normal: *n* = 13, tumor: *n* = 96) was downloaded from the TCGA database and differential analysis was conducted to screen DEmiRNAs using the “edgeR” package (|logFC|>2, *p*adj < 0.01). The target miRNA was confirmed, and the expression position of the target miRNA was localized *via* the EVmiRNA database. Bioinformatics databases including miRDB (http://mirdb.org/), mirDIP (http://ophid.utoronto.ca/mirDIP/index.jsp#r), and TargetScan (http://www.targetscan.org/vert_71/) were employed to predict the target mRNAs of the target miRNA. Besides, ESCC-related mRNA expression data were obtained from TCGA database and differential analysis was carried out to screen differentially expressed mRNAs (DEmRNAs) (|logFC|>1.5, *p*adj < 0.01). Then, the differential mRNA and the predicted target gene were taken to intersect. The ultimate target mRNA that had the binding sites of the target miRNA was obtained. GSEA software was utilized to conduct pathway enrichment analysis on target genes, so as to further investigate the mechanism of target miRNA and its target genes affecting ESCC.

### Patient Samples

This paper included 64 ESCC patients admitted to the Shaoxing People’s Hospital from June 2017 to December 2018. ESCC tissue and adjacent normal tissue samples (within at least 2 cm away from the edge of a tumor) were obtained by surgical resection. All specimens had detailed clinical information, and their tissue samples were confirmed by experts. All patients did not receive radiotherapy, chemotherapy, biotherapy, or traditional Chinese medicine treatment before operation. Peripheral blood was collected from all patients before the operation, and the blood samples of 20 healthy people were also obtained at the same time. This study was authorized by the Hospital Ethics Committee, and written informed consent was acquired from all participants.

### Cell Culture

Normal human esophageal epithelial cell line HEEC and ESCC cell line TE-1 were purchased from BeNa Culture Collection (BNCC, Beijing, China). ESCC cell lines Eca109, EC9706, and NEC were ordered from American Type Culture Collection (ATCC, Manassas, VA, USA). All the above cell lines were cultured in Dulbecco’s Modified Eagle’s Medium (Thermo Fisher Scientific, Waltham, MA, USA) supplemented with 10% fetal bovine serum (Thermo Fisher Scientific, Waltham, MA, USA) routinely. The culture conditions were 37°C and 5% CO_2_ atmosphere. All cells were utilized for subsequent assays after four passages.

### Cell Transfection

The MiRNA-105-5p-mimic, miRNA-105-5p-inhibitor, and their corresponding negative controls (NCs) ordered from GenePharma (Shanghai, China) were transfected into ESCC cells using Lipofectamine 2000 (Thermo Fisher Scientific, Waltham, MA, USA).

The SPARCL1 overexpression vector (oe-SPARCL1) and overexpression negative control (oe-NC) were bought from GenePharma (Shanghai, China). Oe-SPARCL1 and oe-NC were transfected into ESCC cells by the Lipofectamine 2000 reagent kit. After 48 h of cell incubation, the transfection efficiency was tested.

### Separation of EVs in Serum

Serum-derived EVs were purified using the EVs isolation kit (Invitrogen, Carlsbad, CA, United States). In short, reagent and serum samples were subjected to incubation (30 min) and proceeded to centrifugation (10,000 rpm, 5 min, room temperature). Thereafter, precipitated EVs were gathered for resuspending in phosphate-buffered saline (PBS).

### Transmission Electron Microscope Analysis

After EVs were suspended in PBS, a single drop of suspension was dripped in a sample-loaded copperplate. After 1 min of standing at room temperature, the redundant liquid was removed using a piece of filter paper. Subsequently, 2% uranyl acetate (5 μl) was dripped in the sample-loaded copperplate and the samples were negatively stained for 1 min at general temperature. Then, the redundant negative staining reagent was removed. After being dried under an incandescent lamp, the electron microscope H-7600 (Hitachi High-Technologies, Japan) was employed to observe the result, and images were photographed for further analysis.

### Intake of EVs by ESCC Cells

Serum EVs were labeled using the PKH67 Green Fluorescent Cell Linker Mini Kit (Sigma, USA). ESCC cells [10 cells/ml (Sawada et al., 2019)] were plated and cultured for 4 h to foster cell attachment. Subsequently, 2 μl PKH67-labeled EVs were added into the plate and maintained to culture for 48 h. Next, the medium was discarded and the cells were rinsed with PBS and fixed in 4% paraformaldehyde for 10 min to enhance cell permeability. Then, the paraformaldehyde was abandoned and cells were washed with PBS, after which 1 ml 1% bovine serum albumin (BSA) was added and cells were incubated for 20–30 min at room temperature to reduce the staining background. The F-actin and nucleus in the cytoskeleton were stained through adding Alexa Fluor^®^ 594 Phalloidin and the DAPI of appropriate concentration. At last, the intake of EVs in blood-adherent cells by ESCC cells was observed *via* a fluorescence microscope.

### Co-culture of ESCC Cells and Serum-Derived EVs

After being filtered by a 0.22 μm sterilizing filter, serum-derived EVs (20 μg/well) were added into a plate laid with ESCC cells for routine culture in an incubator according to the quantitative results of a bicinchoninic acid kit (Beyotime, China). ESCC cells were isolated for subsequent experiments after 48 h.

### RNA Extraction and qRT-qPCR

For the quantification of miRNA-105-5p, total RNA was extracted from cells, tissue samples, and EVs using RNAiso Plus (TaKaRa, Japan) and QIAzol Lysis Reagent (Qiagen, Hilden, Germany). Then, the total RNA was reverse-transcribed into complementary DNA (cDNA) by using the Superscript II Reverse Transcription Assay Kit (Invitrogen, USA). For detection of SPARCL1 in cells and tissue samples, total RNA was extracted from tissue samples and cells by using TRIzol reagent (TaKaRa, Japan) and transcribed into cDNA by using the M-MLV Reverse transcriptase Assay Kit (TaKaRa, Japan). The concentration of RNA was measured *via* NanoDrop 1000 Spectrophotometer (Thermo Fisher Scientific, USA). qRT-PCR was performed on the ABI 7500 Real-Time PCR system (Thermo Fisher Scientific, USA) using SYBR Prime Script TM RT-qPCR Kit (Takara, Japan). GAPDH and U6 were used as internal controls. The primers were displayed in Table 1. Relative expression levels of miRNA-105-5p and SPARCL1 were analyzed by the 2^−ΔΔCt^ method.

**TABLE 1:** Primer sequences for qRT-PCR.

Genes	Sequences
miRNA-105-5p	Forward: 5′-TCG​GCA​GGT​CAA​ATG​CTC​AGA​C-3′
	Reverse: 5′-CTC​AAC​TGG​TGT​CGT​GGA -3′
U6	Forward: 5′-CTC​GCT​TCG​GCA​GCA​CA-3′
	Reverse: 5′-AAC​GCT​TCA​CGA​ATT​TGC​GT-3′
SPARCL1	Forward: 5′- GCC​TGG​AGA​GCA​CCA​AGA​GGC​C -3′
	Reverse: 5′- ATG​GTC​CCC​AGC​CAA​AAG​CCT​C -3′
GAPDH	Forward: 5′- GAC​CTG​ACC​TGC​CGT​CTA-3′
	Reverse: 5′-AGG​AGT​GGG​TGT​CGC​TGT-3′

### Western Blot

Total proteins were isolated from ESCC cells and EVs using radio-immunoprecipitation assay (Beyotime, China) lysis buffer containing 1% proteinase inhibitor (Beyotime, China). The concentration of the proteins was measured by the BCA protein assay kit (Thermo Fisher Scientific, United States). After denatured at high temperature, protein samples were separated on 10% sodium dodecyl sulfate-polyacrylamide gel electrophoresis (30 μg/lane) and transferred onto polyvinylidene fluoride membranes (Millipore, USA). After being blocked with 5% BSA/Tris-buffered saline with Tween-20 (TBST) for 60 min, the membranes were incubated with primary antibodies overnight at 4°C. On the following day, the membranes were washed with 1 × TBST (Solarbio, China) at room temperature three times with 5 min for each time. Thereafter, the membranes were reacted with horseradish peroxidase-conjugated secondary antibody goat anti-rabbit at room temperature for 120 min, after which the membranes were washed with 1 × TBST in triplicate with 20 min for each time. Finally, the electrochemiluminescence (ECL) assay kit (Solarbio, China) was utilized for protein bands visualization and images were captured for further observation. The antibodies for Western blot were listed in Table 2.

**TABLE 2:** Antibodies for Western blot.

Antibodies	Sources	Dilution multiple	Co. No
TSG101	Rabbit antibody	1:2,000	abcam (ab125011)
CD9	Rabbit antibody	1:2,000	abcam (ab92726)
CD63	Rabbit antibody	1:1,000	abcam (ab217345)
SPARCL1	Rabbit antibody	1:500	abcam (ab107533)
FAK	Rabbit antibody	1:2,000	abcam (ab40794)
*p*-FAK	Rabbit antibody	1:1,000	abcam (ab4792)
Akt	Rabbit antibody	1:500	abcam (ab8805)
*p*-Akt	Rabbit antibody	1:1,000	abcam (ab38449)
GAPDH	Rabbit antibody	1:10,000	abcam (ab181602)
IgG H&L	Goat anti-rabbit	1:10,000	abcam (ab6721)

### Cell Counting Kit-8 Assay

ESCC cells at the logarithmic phase were digested and then 200 μl cells (1 × 10^4^ cells/ml) were seeded in plates for routine incubation. After the cells were cultured for 0, 24, 48, and 72 h, each well was supplemented with 20 μl Cell Counting Kit-8 (CCK-8) solution (Yeasen, Shanghai, China) and the cells were incubated in a constant-temperature incubator for an additional 1 h. At last, the absorbance of each well at 450 nm was identified by SpectraMax M5 (Molecular Devices, USA).

### Wound Healing Assay

ESCC cells at the logarithmic phase were digested, and then 5×10^5^ cells were seeded into plates. After single cell layers were formed, a scratch was made on cells using the tip of a sterile pipette. After being washed with PBS, cells were routinely cultured in FBS-free medium. The cell migration area was photographed at 0 and 48 h under an inverted microscope, and the cell migration rate was calculated as: cell migration rate = (width at 0 h − width at 48 h)/width at 0 h.

### Transwell Invasion Assay

ESCC cells at the logarithmic phase were digested, and 200 μl of cells in an FBS-free medium were then seeded into upper Transwell chambers laid with matrix at a density of 2 × 10^5^ cells/ml. The upper chambers were placed in plates, and the lower chambers were added with a fresh medium containing 10% FBS. After being cultivated at 37°C for 24 h, a soaked cotton swab was used to remove the cells in the upper chambers, after which the cells in the lower chambers were fixed in 4% paraformaldehyde for 10 min and stained with 0.1% crystal violet for 15 min. Five fields were randomly selected, photographed, and counted under an inverted microscope.

### Tumor Formation in Nude Mice

A total of 10 female BALB/C nude mice (4–6 weeks old, weighing 15–20 g) were ordered from the Shanghai Institute of Materia Medica, Chinese Academy of Sciences (Shanghai, China). The mice were divided into two groups with five mice in each group. Approximately 1 × 10^7^ ESCC cells that were diluted by 200 μl PBS were subcutaneously injected into the left hindlimb of nude mice. Tumor volume was monitored every 3 days. Tumor volume was calculated with a formula of: volume= [length × width (Lin et al., 2013)]/2 (mm^3^). When the tumor volume of nude mice reached 100 mm^3^, the serum-derived EVs (20 μg) of ESCC patients were injected into nude mice, with an equivalent amount of PBS being blank control. The injection was performed every 3 days with three consecutive weeks in total. At the end of the third week, the mice were given euthanasia. Then, the tumors were isolated and weighed. The isolated tumors were frozen and stored in liquid nitrogen for further analysis.

### Immunohistochemistry

The tumor tissue of nude mice was fixed in 4% paraformaldehyde and then put into a refrigerator at −4°C. Then, tumor tissue samples were dehydrated in a graded ethanol solution, transparently disposed by xylene, embedded in paraffin, and sliced to about 5 μm slices. Thereafter, the slices were stained with corresponding primary and secondary antibodies, counterstained with hematoxylin, dehydrated, and fixed for further observation. The antibodies for immunohistochemistry were listed in Table 3.

**TABLE 3:** Antibodies for immunohistochemistry.

Antibodies	Sources	Dilution multiple	Co. No
SPARCL1	Rabbit antibody	1:50	abcam (ab125011)
FAK	Rabbit antibody	1:250	abcam (ab40794)
*p*-FAK	Rabbit antibody	1:200	abcam (ab4792)
Akt	Rabbit antibody	1:1,000	abcam (ab8805)
*p*-Akt	Rabbit antibody	1:200	abcam (ab38449)
IgG H and L	Goat anti-rabbit	1:1,000	abcam (ab6721)

### Dual-Luciferase Reporter Gene Assay

The amplified wild-type (WT) and mutant (MUT) 3′UTR of SPARCL1 were cloned into the multiple cloning sites of the pmirGLO luciferase vector (Promega, USA) to generate SPARCL1-WT and SPARCL1-MUT vectors. Using the Lipofectamine 2000 reagent kit, SPARCL1-WT/SPARCL1-MUT vectors and NC-mimic/miRNA-105-5p mimic were co-transfected into ESCC cells. After 48 h of transfection, Firefly and Renilla luciferase activities were evaluated using the Dual-Luciferase Reporter Assay kit (Promega, United States). Each transfection was repeated three times.

### Statistical Analysis

Each assay underwent at least three repetitions. Statistics were processed using GraphPad Prism seven software (La Jolla, CA, USA). Measurement data were exhibited as mean ± standard deviation. The differences between two groups were analyzed by Student’s *t*-test, and one-way analysis of variance was adopted to compare more than two groups. *p* < 0.05 was regarded as statistically significant.

## Results

### MiRNA-105-5p Is Increased in ESCC Tissue and Cells

**FIGURE 1:**
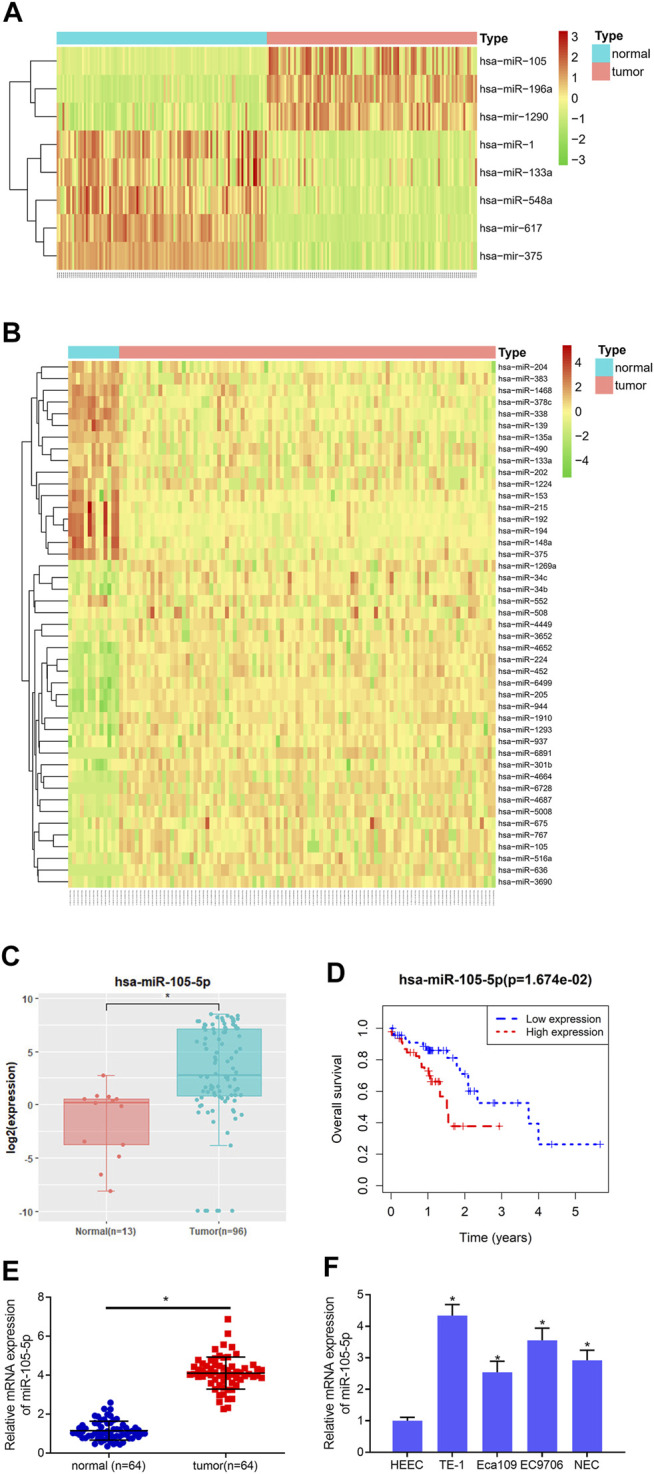
MiRNA-105-5p is activated in ESCC. **(A)** Heatmap for DEmiRNAs in the GSE55856 miRNA chip; **(B)** Heatmap for DEmiRNAs in TCGA-ESCC miRNA dataset; **(C)** MiRNA-105-5p expression in TCGA-ESCC; **(D)** Survival analysis of miRNA-105-5p expression in TCGA-ESCC dataset; **(E)** MiRNA-105-5p expression in cancer and adjacent normal tissue; **(F)** MiRNA-105-5p expression in normal human esophageal epithelial cell line and ESCC cell lines; *p < 0.05.

8 and 45 DEmiRNAs were obtained from the GSE55856 chip and TCGA-ESCC miRNA dataset, respectively, after differential analysis (Figures 1A,B), among which miRNA-105-5p was expressed highly in tumor tissue. Meanwhile, miRNA-105-5p was markedly elevated in tumor tissue in TCGA-ESCC (Figure 1C), and high miRNA-105-5p expression level indicated poor prognosis (Figure 1D). Hence, miRNA-105-5p was chosen. Then, qRT-PCR result suggested that the miRNA-105-5p level was stimulated in ESCC tissue than in normal tissue (Figure 1E). Similarly, cell lines implicated a similar result to tissue: miRNA-105-5p was noticeably increased in ESCC cell lines (Figure 1F) and most highly expressed in the TE-1 cell line. As a result, the TE-1 cell line was chosen for subsequent experiments. Taken together, we confirmed that miRNA-105-5p was highly expressed in ESCC and might be a factor indicating the unfavorable prognosis of ESCC patients.

### MiRNA-105-5p Facilitates ESCC Cell Progression

**FIGURE 2:**
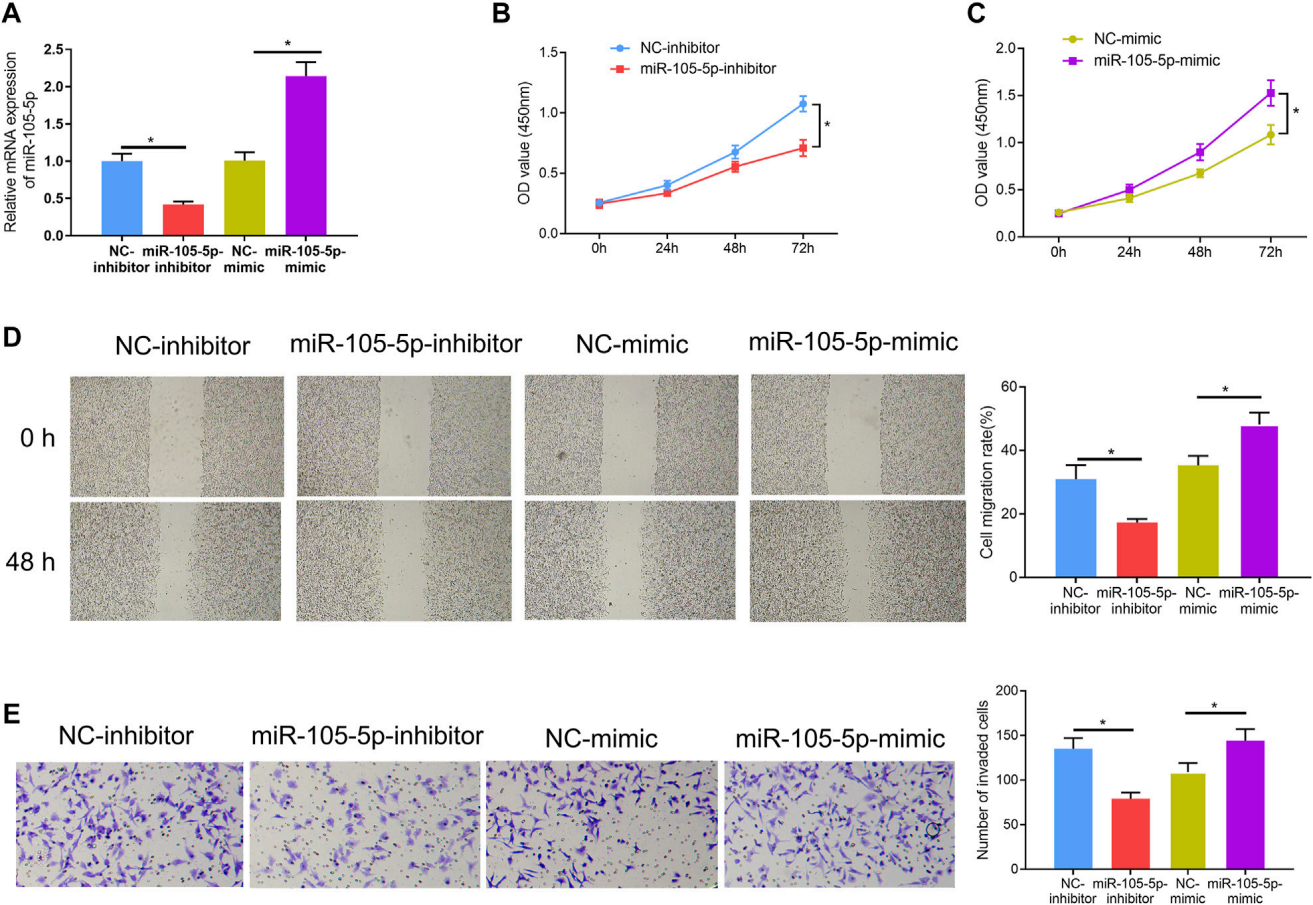
MiRNA-105-5p fosters ESCC cell progression. **(A)** The efficiency of silencing or overexpressing miRNA-105-5p in ESCC cells; **(B,C)** Effect of silencing/overexpressing miRNA-105-5p on cell viability; **(D,E)** The effect of silenced/overexpressed miRNA-105-5p on cell migratory and invasive capacities of ESCC were assessed *via* wound healing assay (40×) **(D)** and Transwell invasion assay (100×) **(E)**, respectively; *p < 0.05.

Since it was discovered that patients with high expression of miRNA-105-5p had poor prognosis, we speculated that miRNA-105-5p was a predictor for the unfavorable prognosis of ESCC. In order to verify this speculation, firstly, miRNA-105-5p-inhibitor/miRNA-105-5p-mimic was transfected into TE-1 cells and the transfection efficiency was confirmed by qRT-PCR (Figure 2A). Then, CCK-8 assay results suggested that ESCC cell viability was reduced/improved upon miRNA-105-5p silencing/overexpression (Figures 2B,C). The results of wound healing assay and Transwell assay uncovered that silencing/overexpressing miRNA-105-5p inhibited/enhanced the cell migration and invasion of ESCC (Figures 2D,E). Collectively, the above experimental results unveiled that miRNA-105-5p facilitated ESCC cell processes and it was likely to be an oncogene of ESCC.

### SPARCL1 is the Direct Target of miRNA-105-5p

**FIGURE 3:**
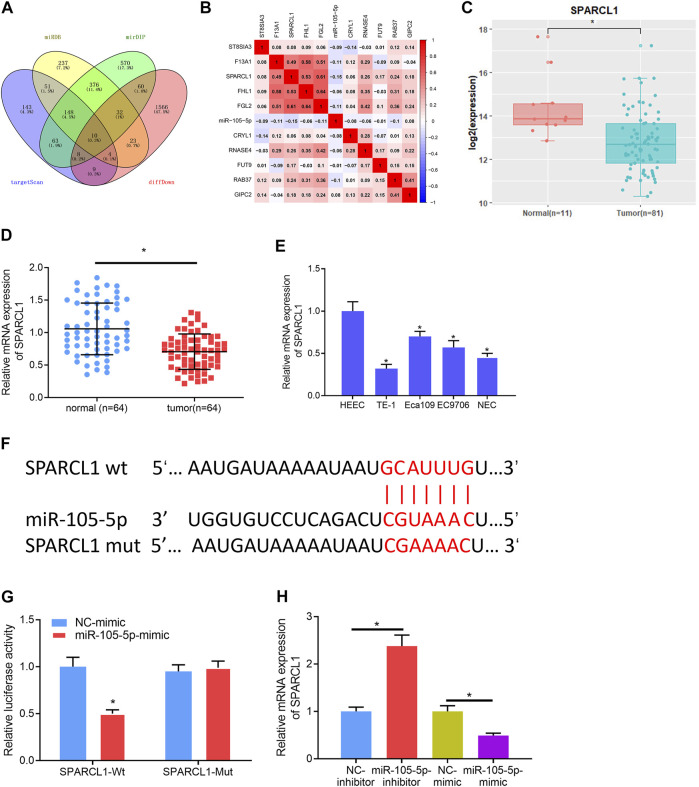
SPARCL1 is the direct target of miRNA-105-5p. **(A)** Venn diagram of downregulated DEmRNAs and the predicted target mRNAs of miRNA-105-5p; **(B)** Correlation of miRNA-105-5p and the 10 overlapping mRNAs; **(C)** SPARCL1 expression in TCGA-ESCC dataset; **(D)** SPARCL1 expression in cancer and adjacent normal tissue; **(E)** SPARCL1 level in normal human esophageal epithelial cell line and ESCC cell lines; **(F)** Putative binding sites between miRNA-105-5p and SPARCL1; **(G)** The targeting relationship between miRNA-105-5p and SPARCL1; **(H)** The effect of silencing/overexpressing miRNA-105-5p in ESCC cells on SPARCL1 level; *p < 0.05, **p < 0.01.

To gain further insight into the mechanism by which miRNA-105-5p modulates ESCC, the downstream mRNA of miRNA-105-5p was explored. Bioinformatics databases including miRDB, mirDIP, and TargetScan were utilized to predict the target mRNAs of miRNA-105-5p, which were overlapped with 1,712 downregulated DEmRNAs from the TCGA-ESCC dataset (Figure 3A). About 10 DEmRNAs that had binding sites with miRNA-105-5p were obtained. Pearson correlation analysis uncovered that SPARCL1 was the strongest negatively correlated with miRNA-105-5p (Figure 3B). Meanwhile, SAPARCL1 was notably decreased in tumor tissue when compared with that in normal tissue (Figure 3C). A qRT-PCR result showed that SPARCL1 was poorly expressed in ESCC cancer tissue and cells, and the expression trend of miRNA-105-5p was opposite (Figures 3D,E). Therefore, we identified SPARCL1 as a potential target of miRNA-105-5p. Firstly, we predicted the binding sequence of miRNA-105-5p and SPARCL1 3′UTR through a bioinformatics database (Figure 3F). WT-SPARCL1 and MUT-SPARCL1 were co-transfected into TE-1 cells with miRNA-105-5p mimic and NC mimic. A Luciferase activity result showed that miRNA-105-5p overexpression decreased the luciferase activity of the WT SPARCL1 reporter but not the MUT reporter, indicating that SPARCL1 was the direct target of miRNA-105-5p (Figure 3G). To further verify the regulatory relationship between miRNA-105-5p and SPARCL1, we transfected miRNA-105-5p inhibitor/mimic into the TE-1 cell line. The result of qRT-PCR showed that SPARCL1 expression was significantly decreased/increased after the overexpression/silence of miRNA-105-5p in TE-1 cells (Figure 3H). Overall, it could be concluded that SPARCL1 was the direct target of miRNA-105-5p.

### MiRNA-105-5p Modulates ESCC Cell Functions by Targeting SPARCL1

**FIGURE 4:**
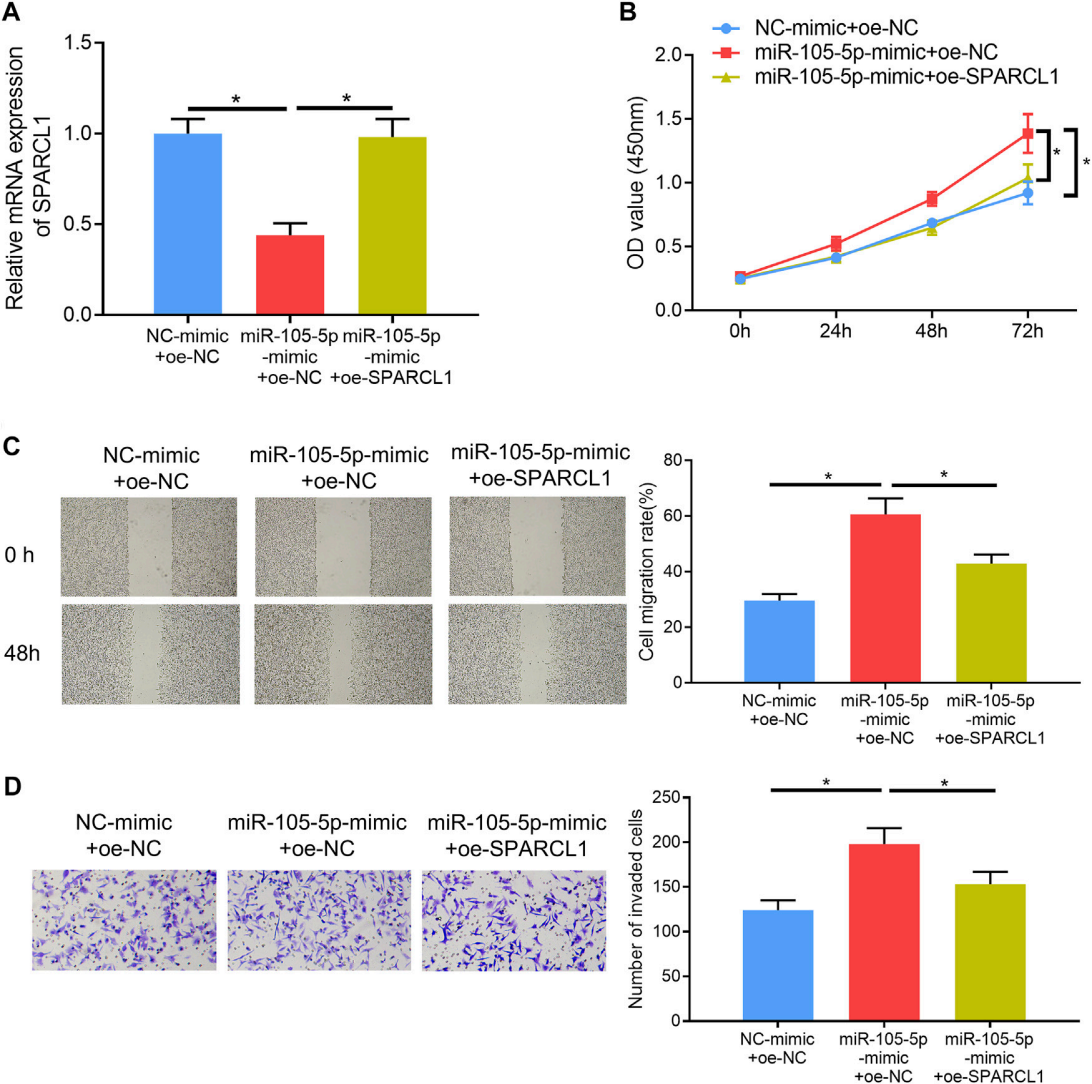
MiRNA-105-5p regulates ESCC cell proliferation, migration, and invasion by targeting SPARCL1. **(A)** SPARCL1 expression in TE-1 cells transfected with NC-mimic + oe-NC, miRNA-105-5p-mimic + oe-SPARCL1, and miRNA-105-5p-mimic + oe-NC; **(B–D)** TE-1 cell proliferation, migration, and invasion in each group were tested *via* CCK-8 assay **(B)**, wound healing assay (×40) **(C),** and Transwell invasion assay (×100) **(D)**, respectively; *p < 0.05.

Rescue experiments were carried out to verify whether miRNA-105-5p could modulate ESCC cell processes *via* mediating SPARCL1. Firstly, miRNA-105-5p and SPARCL1 were overexpressed in TE-1 cells meanwhile, and the qRT-PCR result suggested that SPARCL1 expression in the miRNA-105-5p-mimic + oe-SPARCL1 group was higher than that in the miRNA-105-5p-mimic + oe-NC group (Figure 4A). Subsequently, CCK-8 assay revealed that SPARCL1 suppressed the proliferative ability of ESCC cells and counteracted the promoting effect of miRNA-105-5p on ESCC cell proliferation (Figure 4B). Similarly, wound healing and Transwell invasion assays uncovered that the facilitating effect of miRNA-105-5p on ESCC cell migration and invasion was attenuated by overexpressing SPARCL1 (Figures 4C,D). Collectively, these experiments unveiled that miRNA-105-5p could regulate ESCC cell processes through targeting SPARCL1.

### SPARCL1 Inhibits ESCC Cell Behaviors by Modulating FAK/Akt Signaling Pathway

**FIGURE 5:**
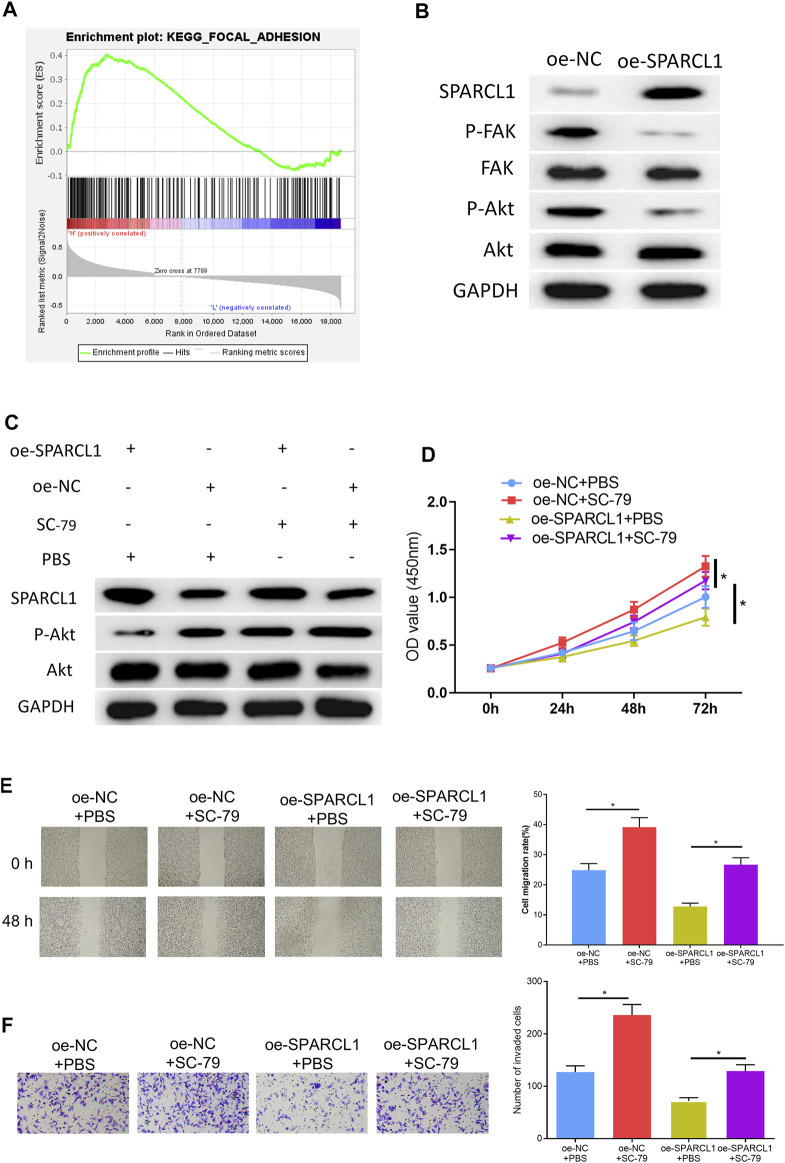
SPARCL1 inhibits ESCC cell progression by modulating FAK/Akt signaling pathway. **(A)** GSEA result of SPARCL1; **(B)** The protein expression levels of SPARCL1 and FAK/Akt signaling pathway-related proteins in ESCC cells upon SPARCL1 overexpression; **(C)** The protein expression levels of SPARCL1 and FAK/Akt signaling pathway-related proteins in ESCC cells with an addition of *p*-Akt activator SC-79; **(D–F)** CCK-8 assay (D), wound healing assay (40×), and **(E)** Transwell invasion assay (×100) **(F)** were carried out to evaluate whether SPARCL1 suppresses ESCC cell proliferation, migration, and invasion *via* FAK/Akt signaling pathway; *p < 0.05.

To observe the SPARCL1-related signaling pathway, we performed GSEA and further discovered that SPARCL1 was markedly enriched in FAK signaling (Figure 5A). Research unveiled that the FAK/Akt signaling pathway is closely associated with ESCC malignant progression (Meng et al., 2016; Zhu et al., 2018). Hence, we examined if there was a connection between SPARCL1 and FAK/Akt signaling. We observed that the levels of phosphorylated FAK and phosphorylated Akt were decreased in SPARCL1-overexpression cell lines, while total FAK and Akt levels remained the same (Figure 5B), which uncovered that SPARCL1 could suppress the activation of the FAK/Akt signaling pathway. Thereafter, ESCC cells with overexpressed SPARCL1 were processed by using *p*-Akt activator SC-79 (10 μM) (Wang et al., 2017) with PBS as control, after which the Western blot result suggested that the activity of the FAK/Akt signaling pathway inhibited by SPARCL1 was reversed by *p*-Akt activator SC-79 (Figure 5C). Finally, tumor-relevant behaviors of ESCC cells were observed in different treatment groups, and finding that adding SC-79 promoted ESCC cell proliferation, migration, and invasion and counteracted the inhibitory effect of SPARCL1 on ESCC cells (Figures 5D–F). Collectively, we demonstrated that SPARCL1 was capable of repressing ESCC cell processes *via* inhibiting the FAK/Akt signaling pathway.

### MiRNA-105-5p in EVs can Be Transferred to ESCC Cells

**FIGURE 6:**
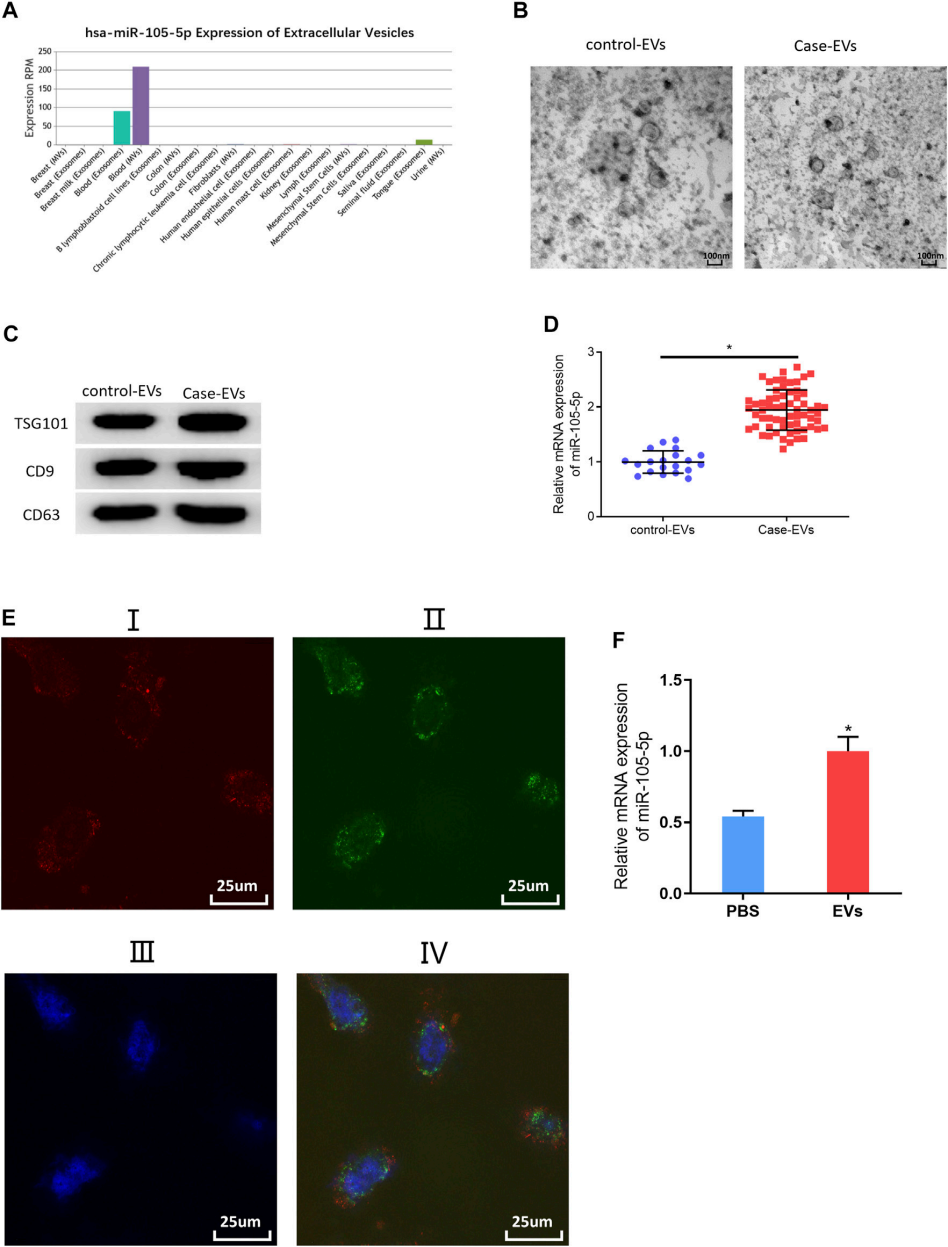
MiRNA-105-5p in EVs can be transferred to ESCC cells. **(A)** Bar chart of miRNA-105-5p expression in different exosomes or microvesicles detected by the EVmiRNA database; **(B)** Transmission electron microscope was used to observe the forms of serum EVs of ESCC patients and normal persons (ratio: 100 nm); **(C)** Protein expression of EVs markers TSG101, CD9, and CD63 in serum EVs of ESCC patients and normal persons; **(D)** MiRNA-105-5p expression in serum EVs of ESCC patients and normal persons; **(E)** A fluorescence microscope was employed to detect whether serum EVs of ESCC patients could be internalized by ESCC cells: I. Alex Fluro594 phalloidin-labeled F-actin (red fluorescence); II. PKH67-labeled microvesicles (green fluorescence); III. DAPI-labeled cell nuclei (blue fluorescence); IV: Merge; Scale bar = 50 nm; **(F)** MiRNA-105-5p expression in ESCC cells after ESCC cells were co-cultured with serum EVs of ESCC patients. *p < 0.05.

Studies indicated that exosomes and EVs could transfer miRNAs that they carry to cancer cells (Zheng et al., 2018; Wan et al., 2020). Accordingly, miRNA-105-5p expression was observed in different exosomes or EVs *via* the EVmiRNA database, finding that miRNA-105-5p mainly existed in serum exosomes and microvesicles, while EVs contained exosomes and microvesicles (Figure 6A). Hence, it was speculated that miRNA-105-5p in EVs could be transferred to ESCC cells. In order to verify this speculation, firstly, transmission electron microscope and Western blot were employed to assess the expression of EV-related proteins (TSG101, CD9, and CD63) to identify that EVs were successfully extracted from blood (Figures 6B,C). Then, the qRT-PCR result revealed that miRNA-105-5p existed in both the serum EVs of ESCC patients (case-EVs) and normal persons (control-EVs), and miRNA-105-5p expression in the EVs of ESCC patients was higher relatively (Figure 6D). Subsequently, Evs of ESCC patients were labeled using PKH67, and ESCC cells were labeled by Alexa Fluor^®^ 594 Phalloidin and DAPI, after which the Evs and ESCC cells were co-cultured with PBS as the blank control. After co-culture was finished, it was confirmed *via* a fluorescence microscope that the EVs of ESCC patients could be internalized by ESCC cells (Figure 6E). At last, qRT-PCR result indicated that miRNA-105-5p was conspicuously increased in ESCC cells (Figure 6F). Taken together, we confirmed that miRNA-105-5p in EVs could be transferred to ESCC cells.

### Serum EVs-Derived miRNA-105-5p of ESCC Patients Promotes ESCC Cell Functions

**FIGURE 7:**
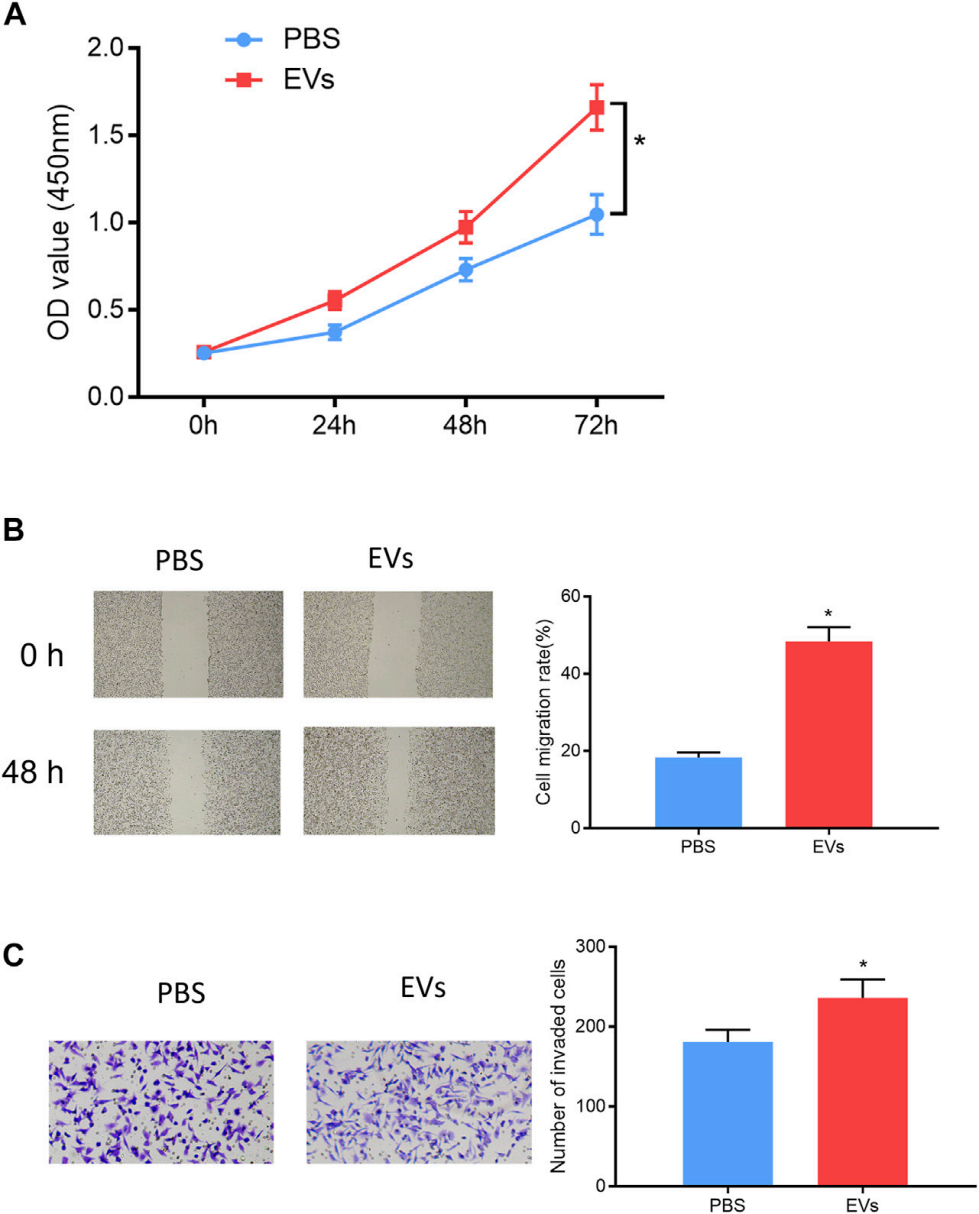
Serum EV-derived miRNA-105-5p of ESCC patients accelerates cell processes. **(A–C)** CCK-8 assay **(A)**, wound healing assay (×40), /**(B)** and Transwell invasion assay (×100) **(C)** were performed to evaluate ESCC cell proliferation, migration, and invasion after ESCC cells were co-cultured with PBS or serum EVs of ESCC patients, respectively; *p < 0.05.

In order to validate that EV-carried miRNA-105-5p could facilitate ESCC cell processes after entering ESCC cells, the ESCC cells that were co-cultured with PBS or serum EVs of ESCC patients were screened, and then cell biological experiments indicated that ESCC cell proliferation, migration, and invasion were noticeably fostered after ESCC cells were co-cultured with the serum EVs of ESCC patients (Figures 7A–C). Collectively, these experiments proved that EVs in the blood could promote ESCC cell progression after being internalized by ESCC cells.

### Serum EVs-Derived miRNA-105-5p Fosters ESCC Cell Growth *In Vivo* Through Targeting SPARCL1 and Regulating FAK/Akt Signaling Pathway

**FIGURE 8:**
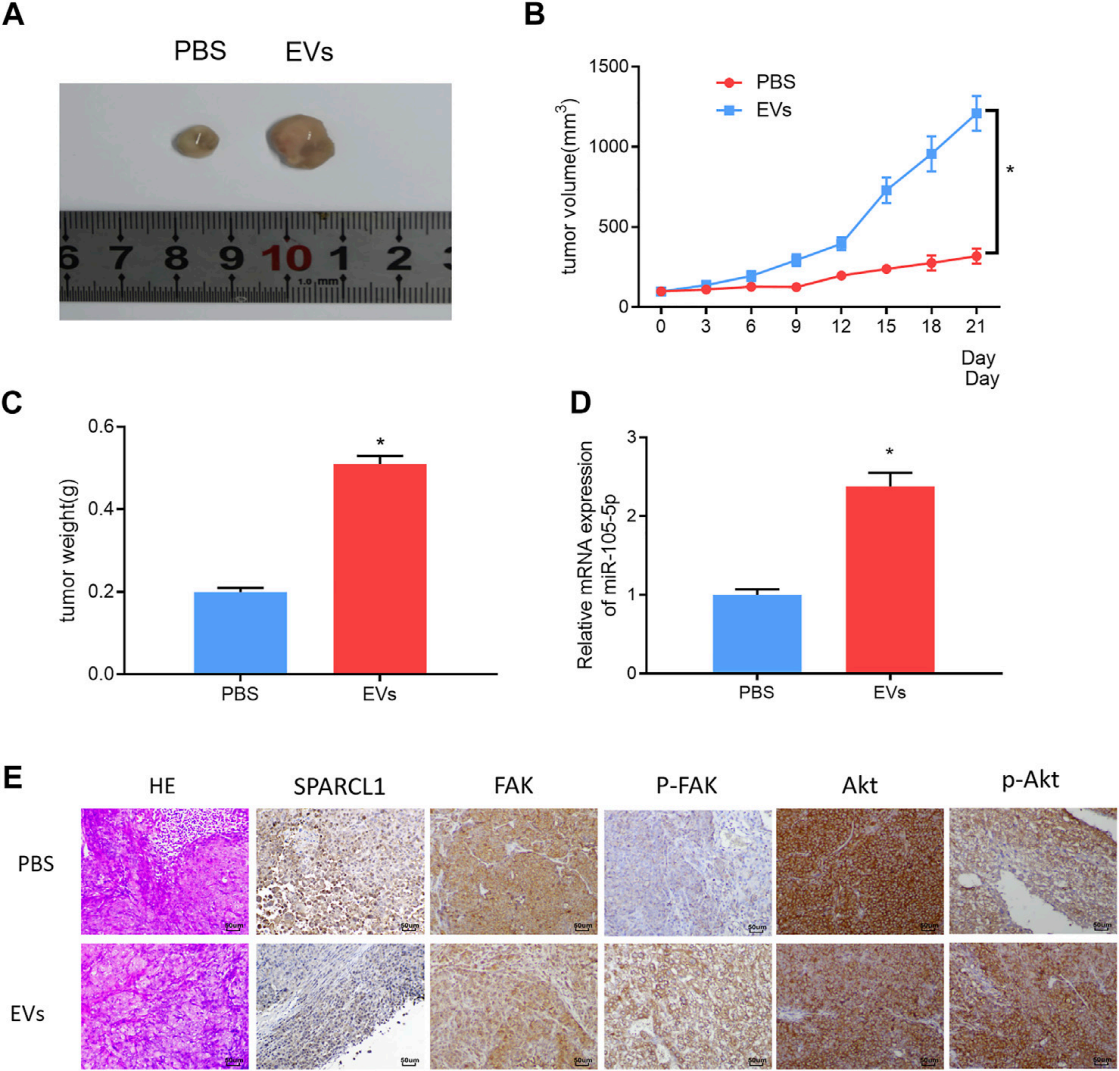
Serum EV-derived miRNA-105-5p fosters ESCC cell growth *in vivo* by targeting SPARCL1 and regulating FAK/Akt signaling pathway. **(A)** Tumors of nude mice that were injected with PBS or serum EVs of ESCC patients; **(B)** Growth curves of tumors of nude mice; **(C)** Tumor weight of nude mice; **(D)** MiRNA-105-5p expression in tumors of nude mice; and **(E)** The protein expression of SPARCL1 and proteins related to FAK/Akt signaling pathway in tumors of nude mice (×200); *p < 0.05.

ESCC cells were subcutaneously injected into the left hindlimb of nude mice. When the tumor volume of nude mice reached 100 mm^3^, the serum EVs of ESCC patients were injected into the tumor of nude mice, with an equal amount of PBS as blank control. The injection was performed every 3 days. Three weeks later, the growth curves of the tumors of nude mice were drawn, and the tumors were weighed. The tumors formed by the serum EVs of ESCC patients grew more rapidly and were larger in size than those of the control group (Figures 8A–C). The qRT-PCR result revealed that miRNA-105-5p upregulation after tumors were injected with the serum EVs of ESCC patients (Figure 8D). Thereafter, immunohistochemistry was carried out to detect the protein levels of SPARCL1, FAK, *p*-FAK, Akt, and *p*-Akt in tumors; the result indicated that the protein expression of SPARCL1 in tumors injected with the serum EVs of ESCC patients was downregulated, whereas those of *p*-FAK and *p*-Akt were upregulated (Figure 8E). Collectively, from these experimental results, we identified that serum EV-derived miRNA-105-5p could foster ESCC cell growth *in vivo* by targeting SPARCL1 and modulating the FAK/Akt signaling pathway.

## Conclusion

Studies have uncovered that the dysregulation of miRNAs can affect the progression of multiple cancers (Chen et al., 2020; Tan et al., 2020; Yehia et al., 2020). Here, it was discovered that miRNA-105-5p was prominently increased in ESCC and was likely to be an unfavorable factor for ESCC prognosis through bioinformatics analysis. A qRT-PCR result implicated that miRNA-105-5p was highly expressed in ESCC. Subsequently, a series of cell biological experiments further proved that miRNA-105-5p could promote the progression of ESCC cells. To sum up, these experimental results validate that miRNA-105-5p is a hopeful molecular target for ESCC treatment.

SPARCL1, also known as Hevin, MAST9, and SCI, is a family member of the SPARC proteins in matricellular proteins (Isler et al., 2001; Bertucci et al., 2004). Research revealed that SPARCL1 was an adhesion molecule mediating the cell–matrix interactions and got involved in physiological processes such as cell proliferation, cell adhesion, muscle differentiation, and B lymphocyte maturation (Bolshakov and Siegelbaum, 1995; Oritani and Kincade, 1998; Claeskens et al., 2000; Maak et al., 2001). With the research on SPARCL1 going deeper in recent years, it has been found to play a vital role in affecting progression of cancers through being regulated by miRNAs. For instance, targeting SPARCL1 by miRNA-539-3p facilitates the progression of epithelial ovarian cancer cells (Gong and Fan, 2019). Nevertheless, the role of SPARCL1 in ESCC has not been reported yet. Herein, it was discovered that miRNA-105-5p had binding sites with SPARCL1 through bioinformatics methods. Given this finding, firstly, SPARCL1 expression in ESCC tissue and cell lines was detected, finding that SPARCL1 was poorly expressed. Meanwhile, dual-luciferase reporter assay and qRT-PCR confirmed that miRNA-105-5p could target SPARCL1 and regulate its expression. Additionally, a series of *in vitro* experiments validated that miRNA-105-5p was capable of fostering ESCC cell progression *via* mediating SPARCL1.

FAK is located in the focal adhesion that forms between cells growing with extracellular matrix constituents. FAK can activate the FAK/Akt signaling pathway *via* receiving signals from integrin, fibrin, etc. Activated FAK can combine with Scr to form a complex, and the Tyr397 of activated FAK can directly combine with the SH2 domain of P13K to activate P13K. Activated P13K is capable of activating Akt so as to regulate cell growth (Gan et al., 2006). Currently, loads of studies have reported that mRNA can regulate ESCC cell proliferation and metastasis *via* the FAK/Akt signaling pathway. For example, LOXL2 fosters the tumorigenesis of head and neck squamous cell carcinoma through FAK/Akt signaling (Liu et al., 2020). Here, the GSEA result implicated that SPARCL1 was enriched in the FAK/Akt signaling pathway. Based on this, the Western blot result suggested that SPARCL1 was able to suppress the FAK/Akt signaling pathway. Thereafter, ESCC cells with SPARCL1 overexpression were processed using *p*-Akt activator, and cell biological experiments were then conducted to evaluate ESCC cell behaviors. Taken together, we proved that SPARCL1 was capable of repressing the progression of ESCC cells through deactivating the FAK/Akt signaling pathway.

Quite a few studies have demonstrated that EVs can be used to treat tumors in recent years (Zheng et al., 2017; Wang et al., 2019). Therefore, miRNA-105-5p expression was observed *via* the EVmiRNA database, finding that miRNA-105-5p mainly existed in serum exosomes and Evs. Then, the serum Evs of ESCC patients and normal persons were obtained. qRT-PCR revealed that miRNA-105-5p existed in blood and that the serum Evs of ESCC patients could be internalized by ESCC cells, leading to elevated miRNA-105-5p expression in ESCC cells, ultimately facilitating ESCC cell behaviors. Subcutaneous transplantation xenograft models are widely applied to the study on the treatment of tumors with miRNAs in Evs (Wang et al., 2020; Zhao et al., 2020). In this study, it was also confirmed that serum EV-derived miRNA-105-5p could promote tumor growth by employing subcutaneous transplantation tumor models in nude mice. Collectively, the above experimental results fully verified that serum EV-derived miRNA-105-5p can be transferred to ESCC cells to foster the tumorigenesis of ESCC.

Altogether, we demonstrated that serum EV-derived miRNA-105-5p could be transferred to ESCC cells and foster the progression of ESCC by targeting SPARCL1 and regulating the FAK/Akt signaling pathway. The discovery of this functional mechanism will supply a rationale for ESCC therapy. Furthermore, how miRNA-105-5p regulates the expression of other target genes in ESCC or in a broad scope of other cancers remains to be further studied in the near future. This study also had certain defects, like this study carried out cell function verification only using one cell line, and we will use other ESCC cell lines to further verify the results of this study.

## Data Availability

The original contributions presented in the study are included in the article/Supplementary material, further inquiries can be directed to the corresponding authors.
